# Tongqiaohuoxue Hinders Development and Progression of Atherosclerosis: A Possible Role in Alzheimer’s Disease

**DOI:** 10.3390/biology9110363

**Published:** 2020-10-27

**Authors:** Eunyoung Ha, Mikyung Kim, Jaemoo Chun, Chang-Seob Seo, YouMee Ahn, Jeeyoun Jung

**Affiliations:** 1 Department of Biochemistry, School of Medicine, Keimyung University, Daegu 42601, Korea; hanne.md@gmail.com (E.H.); balee96@naver.com (M.K.); 2 Non-Clinical Research Collaboration Team, Korea Institute of Oriental Medicine, Daejeon 34054, Korea; jchun@kiom.re.kr; 3 Herbal Medicine Research Division, Korea Institute of Oriental Medicine, Daejeon 34054, Korea; eyoung43@daum.net; 4 Clinical Medicine Division, Korea Institute of Oriental Medicine, Daejeon 34054, Korea; nescafe.xo@gmail.com

**Keywords:** Tongqiaohuoxue decoction, atherosclerosis, apolipoprotein E, Alzheimer’s disease, resistin

## Abstract

**Simple Summary:**

Alzheimer’s disease and coronary heart disease are two ever-increasing major health concerns worldwide. Scientific studies revealed a link between Alzheimer’s disease and atherosclerosis, a major causality of coronary heart disease. Herbal medicine has been widely prescribed to treat Alzheimer’s disease and atherosclerosis. In the current study, we explored the possible therapeutic effect of Tongqiaohuoxue, a herbal medicine developed during the Qing dynasty of China for the prevention and treatment of cardiovascular disease, on Alzheimer’s disease and atherosclerosis. We discovered Tongqiaohuoxue showed therapeutic effects not only on atherosclerosis but also on Alzheimer’s disease. Tongqiaohuoxue treatment into the animal model of Alzheimer’s disease and atherosclerosis attenuated atherosclerotic plaque and brain amyloid formations, abnormalities that are characteristic of coronary heart disease and Alzheimer’s disease, respectively. Based on these findings, Tongqiaohuxue showed promising therapeutic effects for the treatment of patients with both Alzheimer’s disease and coronary heart disease.

**Abstract:**

Atherosclerosis is closely associated with Alzheimer’s disease (AD). Tongqiaohuoxue decoction (THD) is a classical herbal prescription in traditional Chinese medicine widely used for the prevention and treatment of cerebrovascular disease. This study aimed to explore the therapeutic effects of THD on atherosclerosis and AD. Eight-week-old C57BL6/J wild-type and ApoE-deficient (ApoE-/-) mice were fed a high-fat and high-cholesterol diet for eight weeks, followed by oral phosphate-buffered saline vehicle or THD treatment for eight weeks further. In ApoE-/- mice, THD attenuated lipid deposition in the aorta and the brain, and abrogated atherosclerotic changes without affecting serum lipid profiles while decreasing amyloid plaque formation. In vitro assays undertaken to understand THD’s effects on lipid clearance in the aorta and brain vessels revealed that THD treatment inhibited the lipid uptake, stimulated by oxidized low-density lipoprotein, resulted in decreased endothelial cell activation through reduction in intercellular adhesion molecule-1, vascular cell adhesion molecule-1, and monocyte chemoattractant protein-1 levels. Serum analysis revealed inhibitory effects of THD on resistin production, which has important roles in the development of both atherosclerosis and AD. In conclusion, the current study demonstrates beneficial effects of THD on the development and progression of atherosclerosis, and a possible protective role against AD.

## 1. Introduction

Increasing evidence suggests a link between Alzheimer’s disease (AD), vascular risk factors, and atherosclerosis in elderly patients [1]. Atherosclerosis has been suggested to play a role in cognitive deterioration in the elderly [2,3], and systemic atherosclerosis treatment could thus potentially remediate AD [4].

In particular, apoprotein E (ApoE) is a polymorphic protein whose primary functions are to transport lipids and to participate in lipoprotein and cholesterol metabolism; it has been reported as a risk factor for both atherosclerosis and AD [5]. The e4 variant of the ApoE gene exhibits as a modest genetic risk factor for atherosclerosis [6] and is the most important genetic risk factor for sporadic AD in the general population [7]. Intriguingly, a recent study revealed the complement-regulating function of ApoE, directly linking ApoE to the pathogenesis of AD [8].

Tongqiaohuoxue decoction (THD; known as Tonggyuhwalhyeol-tang in Korea) was developed during the Qing dynasty of China (A.D. 1830). It is widely used in traditional East Asian medicine, particularly in China, Japan, and Korea, for the prevention and treatment of cerebrovascular disease [9]. The evidence indicated that THD protected neuronal cells from glutamate-induced toxicity in vitro [9,10] and ameliorated learning and memory defects in rats with vascular dementia [11]. In addition, a recent study demonstrated the efficacy and functional mechanism of THD in mice with high-fat diet-induced obesity, showing metabolic dysregulation, inflammation, and a prothrombotic state, as an early vascular model [12]

Given the involvement of ApoE in both atherosclerosis and AD, and the aforementioned evidence of neuronal and/or vascular protective actions of THD, we hypothesized that THD has a therapeutic effect on AD and atherosclerosis. Therefore, in the current study, we aimed to assess the efficacy of THD in the treatment of atherosclerosis and explore its use as a therapeutic drug in the prevention and treatment of ApoE-related brain disease, especially AD, using ApoE-deficient mice fed with a high-fat and high-cholesterol (HFHC) diet.

## 2. Materials and Methods

### 2.1. Chemicals and Reagents

We used 11 standard reference compounds for quality assessment of THD—gallic acid (99.5%), amygdalin (99.0%), albiflorin (99.8%), paeoniflorin (99.4%), ferulic acid (98.0%), safflomin A (99.7%), benzoic acid (99.9%), benzoylpaeoniflorin (98.0%), 6-gingerol (99.3%), costunolide (98.0%), and dehydrocostus lactone (98.0%) (Merck KGaA, Darmstadt, Germany; Wako Chemicals, Osaka, Japan; Chem Faces Biochemical Co., Ltd., Wuhan, China; Shanghai Sunny Biotech Co., Ltd., Shanghai, China). High-performance liquid chromatography (HPLC)-grade solvents (methanol, acetonitrile, and water) and ACS reagent (formic acid for HPLC, ≥98.0%) were purchased from J.T. Baker (Phillipsburg, NJ, USA), and Merck KGaA (Darmstadt, Germany), respectively. Donepizil hydrochloride was purchased from Merck KGaA (Darmstadt, Germany).

### 2.2. HPLC Analysis of THD

Simultaneous determination of the 11 marker components for THD quality control was performed using the LC-20A Prominence HPLC system (Shimadzu Co., Kyoto, Japan) coupled with a photo-diode array (PDA) detector and Lab Solution software (version 5.53, SP3, Kyoto, Japan). Analyte separation was carried out using the Sun Fire C18 analytical column (4.6 × 250 mm, 5 μm; Milford, MA, USA), constantly maintained at 40 °C. The mobile phase for the efficient separation and analysis of the marker components in the THD sample consisted of distilled water (A) and acetonitrile (B), both containing 0.1% (*v/v*) formic acid. The gradient eluting conditions were as follows: 0–30 min, 5–60% B; 30–40 min, 60–100% B; 40–45 min, 100% B; 45–50 min, 100–5% B. For the analysis, 200 mg of the lyophilized THD sample was dissolved in 20 mL of distilled water, and the solution was extracted using an ultrasonicator (Branson 8510E-DTH, Denbury, CT, USA) for 60 min. The extracted solution was filtered using a 0.2-μm membrane filter (PALL Life Sciences, Ann Arbor, MI, USA) before sample injection for HPLC analysis.

### 2.3. THD Preparation

All herbal plants were purchased from Omniherb (Daegu, Korea) and THD was prepared as previously described (Kim et al., 2016). In brief, 216 g of a mixture of eight dried plants [16 g *Paeonia obovata Maxim*, 16 g *Ligusticum officinale* (Makino) Kitag., 48 g *Prunus persica* (L.) Batsch, 48 g *Carthamus tinctorius* L., 12 g *Allium fistulosum* L., 8 g Ziziphus jujuba var. inermis (Bunge) Rehder, 48 g *Zingiber officinale Roscoe*, and 20 g *Aucklandia costus* Falc.] was boiled in distilled water for 2 h at 100 °C, followed by filtration through a 0.2-μm membrane filter. The filtrates were then freeze-dried and stored at −70 °C. The yield was 20.02%. The THD extract was dissolved in drinking water for oral administration. A voucher specimen (# BS-7) was deposited at the Korea Institute of Oriental Medicine and a list of the full taxonomic names of all species used in this study is presented in Table S1 (Supplementary Materials).

### 2.4. Animals

All experimental procedures were approved by the experimental Animal Care Committee at the Keimyung University, School of Medicine (KM-2018-09) and were performed according to their guidelines. Male ApoE-deficient mice (ApoE-/-; C57BL/6J background, 6-week-old) and C57BL/6J wild-type (WT) mice were purchased from The Jackson Laboratory (Sacramento, CA, USA). Both types of mice were fed a high fat, high cholesterol (HFHC) diet (D12109C, Research Diets, New Brunswick, NJ, USA) for 8 weeks. The ApoE-/- mice were then randomly divided into two groups [control (*n* = 10) and THD-treated (*n* = 10)] and received either daily oral phosphate-buffered saline (PBS) or THD (100 mg/kg) treatment for additional 8 weeks. Mice were sacrificed at the end of the experimental period. Serum samples were harvested and centrifuged at 2000× *g* for 10 min. Brain and aortic tissues were fixed and processed for histological analyses. The remaining tissues were quickly frozen in liquid nitrogen and stored at −80 °C for further analysis.

### 2.5. Evaluation of Atherosclerosis

Atherosclerotic lesions and lipid deposition in the aorta were analyzed. Briefly, to obtain a flat preparation, the fixed aorta was cut longitudinally from the arch to the iliac bifurcation. The aorta was then stained with oil red O (ORO; 0.5% *w/v* in isobutanol), followed by washing. The aortic tissues were examined under a light microscope (Leica, Morrisville, NC, USA). The root of the aorta was stained with hematoxylin and eosin (H&E). Similarly, brain tissues were embedded and frozen in optimal cutting temperature (OCT) compound at −80 °C. Twenty-micrometer mouse whole-brain coronal sections were prepared using a MEV cryotome (Slee medical GmbH, Mainz, Germany) and stained using ORO.

### 2.6. Lipid Profiles

Blood samples were harvested into tubes and centrifuged at 1000× *g* force for 10 min to obtain sera. Total cholesterol, low-density lipoprotein (LDL)-cholesterol, and high-density lipoprotein (HDL)-cholesterol levels in the serum were determined using kits from BioVision Ltd. (Milpitas, CA, USA), in accordance with the manufacturer’s instructions. The triglyceride levels were determined using a Triglyceride Colorimetric Assay Kit (Cayman Chemical, Ann Arbor, MI, USA). All experimental procedures were executed according to the manufacturer’s instructions. The values of the absorbance were determined using an ELISA microplate reader (Biochrom, Cambridge, UK).

### 2.7. Cell Culture

Human THP-1 monocyte cell line (ATCC^®^ TIB-202TM) was obtained from ATCC (Manassas, VA, USA). Cells were maintained in RPMI-1640 medium supplemented with 10% heat-inactivated fetal bovine serum (GIBCO, Thermo Fisher Scientific Inc., Waltham, MA, USA), 100 U/mL penicillin, and 100 μg/mL streptomycin (GIBCO, Thermo Fisher Scientific Inc., Waltham, MA, USA). Cells were cultured in 5% CO_2_-humidified atmosphere at 37 °C and passaged twice a week. THP-1 cells were differentiated by addition of phorbol myristate acetate (Sigma-Aldrich, St. Louis, MO, USA) to the culture medium at a final concentration of 100 nM for 24 h. Human umbilical vein endothelial cells (HUVECs) were purchased from LONZA Biologics (LONZA Biologics, Cambridge, UK), and maintained in Endothelial Basal Medium-2 supplemented with 2% FBS and growth factors. Cells were incubated in a humidified, 37 °C incubator under 5% CO_2_. Cell passages between 3 and 5 were used in the present study.

### 2.8. LDL Isolation and Oxidation

LDL was isolated from voluntary human subjects recruited from Keimyung University Dongsan medical center from all of whom informed consents were obtained. All procedures were approved by the ethics committee of Keimyung University, School of Medicine (KM-2018-09). Blood samples were collected into sterile EDTA tubes and the plasma was separated by centrifugation at 2000× *g* for 20 min. LDL was isolated by sequential ultracentrifugation at a final density of 1.019–1.063 g/mL with potassium bromide in EDTA-saline, followed by dialysis in 1 mM EDTA buffer (pH 8.0) before oxidation. The isolated LDL was oxidized by incubation of 0.2 mg of LDL protein/mL for 4 h at 37 °C with 5 μM CuSO4 (Sigma-Aldrich, St. Louis, MO, USA) in PBS (pH 7.4). The oxidized LDL (oxLDL) was then dialyzed for 36 h at 4 °C in 0.15 M NaCl solution containing 0.01% EDTA buffer (pH 7.0).

### 2.9. Lipid Uptake Assay

THP-1 cells (3.5 × 105) were seeded on rounded cover slips in Costar^®^ 24-well tissue culture plates (Corning, Corning, NY, USA) and treated with phorbol 12-myristate 13-acetate (PMA, 100 nM; Sigma) in growth medium. After differentiation, the cells were incubated with oxLDL (200 μg/mL) for 24 h. Lipid uptake by macrophages was quantified by ORO staining.

### 2.10. ORO Staining

THP-1 cells were fixed with 4% paraformaldehyde (PFA; Fujifilm Wako Chemicals, USA, Richmond, VA, USA) for 1 h. A stock solution of ORO (0.5% in isopropanol) was diluted and filtered (60% stock solution and 40% distilled water). Cover slips were stained with ORO for 20 min and counterstained with Mayer’s hematoxylin (Dako North America Inc., Carpinteria, CA, USA) for 1 min and then dried and mounted using an aqueous mounting medium. Positively stained (red) cells were identified as macrophage-derived foam cells, which were observed under a light microscope (Leica, Wetzler, Germany).

### 2.11. Monocyte Adhesion to Endothelial Cells

THP-1 cells were labeled with the fluorescent molecule BCECF-AM (5 μmol/L, Abcam, UK) for 30 min at 37 °C, followed by washing twice with phosphate-buffered saline (PBS) and resuspension in RPMI-1640 medium. HUVECs were seeded in an eight-well chamber (Thermo Fisher Scientific, USA) and were pre-treated with 100 μg/mL of THD for 1 h. The confluent HUVEC monolayer was then stimulated with 100 μg/mL of oxLDL and incubated with BCECF-AM-labeled THP-1 cells (2 × 105/well) in RPMI-1640 medium containing 10% FBS at 37 °C for 30 min. Unbound monocytes were subsequently removed by washing twice with warm PBS.

### 2.12. Quantitative Real-Time PCR

Total RNA was isolated from cells using RNeasy kit (Qiagen, Cologne, Germany). Reverse transcription was performed to yield cDNA using the High-Capacity cDNA Reverse Transcription Kit (Applied Biosystems, Foster City, CA, USA). The RNA (1 μg) was performed using Sso Advanced Universal SYBR Green Supermix (Bio-Rad, Hercules, CA, USA) on CFX Connect Real-Time System (Bio-rad, Hercules, CA, USA). GAPDH was used as an internal control.

### 2.13. Analysis of Adipokine, BACE1 and AChE

The plasma adipokines levels were analyzed using the Bio-Plex Pro Mouse Diabetes Set immunoassay kit (Bio-Rad Laboratories, Hercules, CA, USA). Inhibition of AD related enzyme including ß-secretase and acetylcholinesterase was evaluated using SensoLyte^®^ 520 BACE1 assay kit (AnaSpec, Fremont, CA, USA) and AChE assay kit (ab138871, abcam, Cambridge, UK) respectively, according to the manufacturer’s instructions.

### 2.14. Immunohistochemistry

Congo red staining was performed to detect Aβ aggregation. Briefly, after the −80 °C incubation, slides were fixed with 4% paraformaldehyde for 10 min and then washed under tap water. The sections were stained with Congo red (1% *w/v*), differentiated with alkaline alcohol (1% potassium hydroxide in 80% ethanol), and counterstained with hematoxylin for 4 min before they were dehydrated and mounted. Plaques were observed under a light microscope.

### 2.15. Statistical Analysis

All data were analyzed using Prism software, version 8.0 (GraphPad Software, San Diego, CA, USA). Values are presented as mean ± standard deviation (S.D.) Statistical significance was determined based on *p*-values < 0.05 obtained from one-way ANOVA with Tukey’s post- hoc tests.

## 3. Results

### 3.1. Chemical Components in THD

Eleven constituents—gallic acid, albiflorin, paeoniflorin, benzoic acid, and benzoylpaeoniflorin from *Paeoniae radix*; ferulic acid from *Cnidii rhizoma* and *Allii fistulosi bulbus*; amygdalin from Persicae semen; safflomin A from *Carthami flos*; 6-gingerol from *Zingiberis rhizoma recens*; and costunolide and dehydrocostus lactone from *Aucklandiae radix*—were determined as marker components for quality control of the THD sample. Simultaneous analysis of these marker analytes was carried out using the HPLC–PDA method.

**Figure 1:**
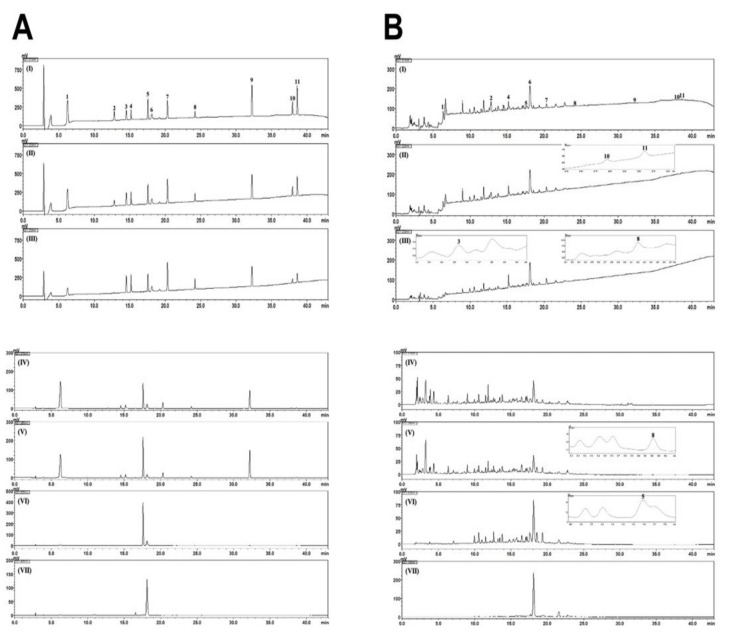
HPLC chromatograms of the standard mixture (**A**) and THD sample (**B**) at UV detection wavelengths of 214 nm (**I**), 220 nm (**II**), 230 nm (**III**), 270 nm (**IV**), 280 nm (**V**), 320 nm (**VI**), and 400 nm (**VII**). Gallic acid (1), amygdalin (2), albiflorin (3), paeoniflorin (4), ferulic acid (5), safflomin A (6), benzoic acid (7), benzoylpaeoniflorin (8), 6-gingerol (9), costunolide (10), and dehydrocostus lactone (11).

Using this optimized analytical method, all marker analytes were eluted within 40 min with a resolution >2.3 (Figure 1). In all analytes, the coefficient of determination (r2) of the regression equation for quantification was 0.9999 and 1.000, suggesting very good linearity at the tested concentration levels (Supplementary Materials, Table S2). The amounts of the eleven marker components ranged from 0.08 to 7.44 mg/lyophilized g (Supplementary Materials, Table S3). Details regarding chemical components in THD are mentioned in Supplementary results.

### 3.2. Reduction of Atherogenic Plaque Formation with THD Treatment in ApoE-/- Mice

**Figure 2:**
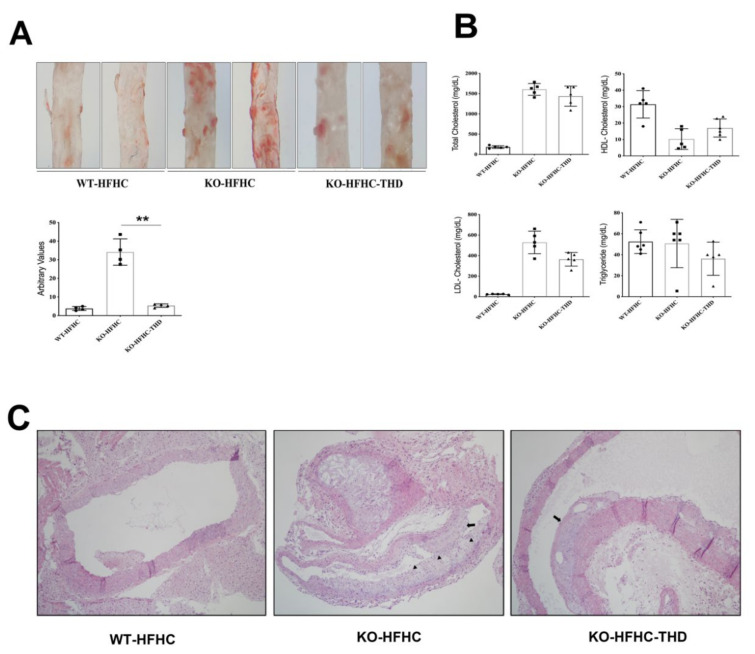
THD attenuates atherosclerotic changes in ApoE-/- mice. (**A**). Upper panel; Oil red O staining of the aorta. Magnification, ×4. Lower panel; Quantification of the Oil red O-stained area. (**B**) Serum lipid profiling. (**C**) Hematoxylin-eosin (H and E) staining of aortic roots. Magnification, ×10. WT-HFHC, wild type–high fat high cholesterol; KO-HFHC, ApoE-/- high fat high cholesterol; KO-HFHC-THD, ApoE-/- high fat high cholesterol–tongqiaohuoxue decoction. Arrows show: fatty streaks, Arrow heads show: foam cells, ** *p* < 0.001 vs. KO-HFHC. *p* value was obtained from one-way ANOVA with Tukey’s post- hoc tests.

Plaque formation is one of the most important phenotypic characteristics of atherosclerosis. The ApoE-/- mice fed a HFHC diet showed more atherosclerotic Oil red O staining (ORO)-positive plaques in aortic regions than did the WT-HFHC control mice (Figure 2A). In contrast, treatment with THD significantly reduced aortic wall plaque deposits in the ApoE-/- mice to levels close to those of the WT-HFHC control mice (*p* < 0.001). Lipid profile analysis showed no difference in the levels of total cholesterol, high-density lipoprotein (HDL)-cholesterol, and low-density lipoprotein (LDL)-cholesterol, and triglycerides between ApoE-/- mice with and without THD treatment (Figure 2B).

We next examined H&E staining for atherosclerotic microanatomical changes in all experimental aortic tissues (Figure 2C). ApoE-/- mice fed with HFHC exhibited increased foam cells and plaque formation blocking the lumen of the aortic vessels. THD treatment attenuated the deposition of foam cells, plaque formation, and macrophage infiltration to the adventitia of the aorta of ApoE-/- HFHC mice.

### 3.3. Attenuation of HFHC-Induced Lipid Deposition and amyloid-beta (Aβ) Plaque Formation in the Brains of ApoE-/- Mice

**Figure 3:**
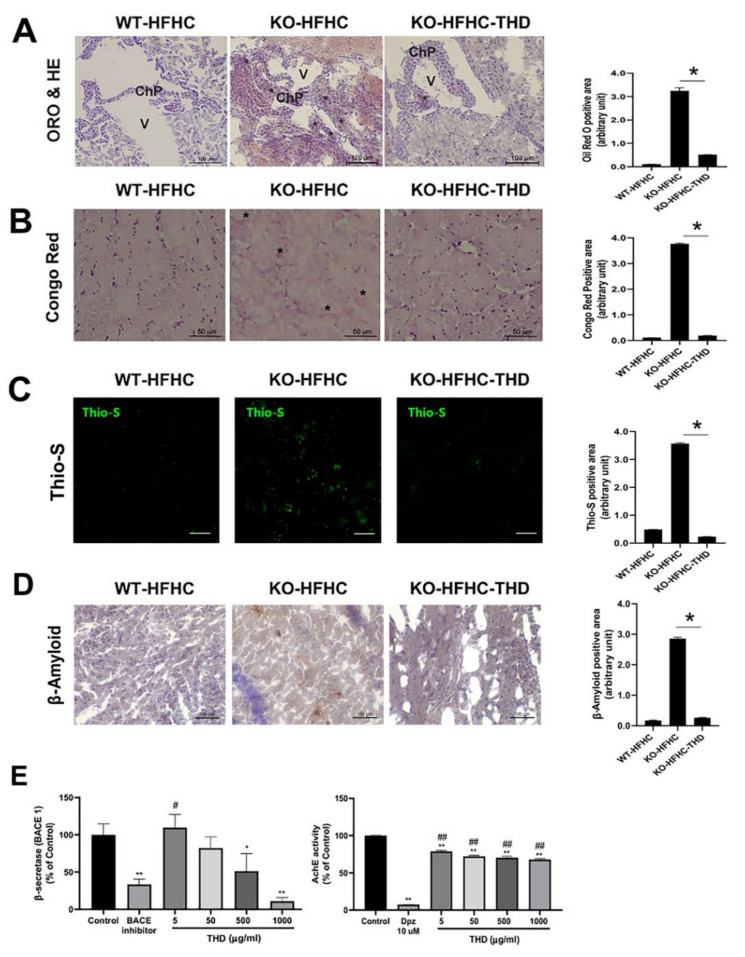
THD attenuates lipid accumulation and amyloid plaque formation in the brain of ApoE-/- mice. (**A**) Left panel; Oil red O staining of the brain, Right panel; Quantification of the Oil red O stained area. (**B**) Left panel; Congo red staining of the brain, Right panel; Quantification of the Congo red stained area. (**C**) Left panel; Thioflavin-S (Thio-S) staining of the brain, Right panel; Quantification of the Thio-S stained area Scale bars = 20 μm). (**D**) Left panel; β-amyloid staining of the brain, Right panel; Quantification of the β-amyloid stained area. (**E**) Effect of THD on ß-secretase (BACE1) acetylcholinesterase (AChE) activity. Asterisk indicates positive staining for oil red O in (**A**) and Congo red in (**B**). Data in (**E**) are expressed as percentages of the corresponding vehicle control (mean ± SD), *n* = 4. * *p* < 0.01, ** *p* < 0.001, vs. the corresponding control. # *p* < 0.01, ## *p* < 0.001 vs. the corresponding inhibitor. *p* value was obtained from one-way ANOVA with Tukey’s post- hoc tests. WT-HFHC, wild type–high fat high cholesterol; KO-HFHC, ApoE-/- high fat high cholesterol; KO-HFHC-THD, ApoE-/- high fat high cholesterol–tongqiaohuoxue decoction; ChP, choroid plexus; V, ventricle; Dpz, donepezil hydrochloride.

Given the pathophysiological role of ApoE in atherosclerosis and AD, we next investigated the blood–cerebrospinal fluid (CSF) barrier of the choroid plexus (ChP) region in ApoE-/- mice to determine the neuroprotective effects of THD. HFHC appeared to stimulate deposition of lipids in the ChP region of ApoE-/- mice, whereas the effect was attenuated with THD treatment (Figure 3A). We next examined Aβ accumulation using Congo red staining. As shown in Figure 3B, ApoE-/- mice fed with HFHC showed increased deposition of Aβ in the pars compacta of the substantia nigra region, whereas treatment with THD showed a substantially lower accumulation (Figure 3B). We also validated inhibitory effect of THD on the Aβ accumulation via thioflavin-S immunofluorescence and β-amyloid immunohistochemistry staining (Figure 3C,D).

In addition, THD inhibited the activation of AD related enzyme including β-secretase (BACE 1) and acetylcholinesterase (AchE), which are required for the production of neurotoxic Aβ and the promotion of Aβ fibril formation, respectively [13]. THD decreased the levels of BACE 1 in a dose-dependent manner. In particular, 500 μg/mL (*p* = 0.006) and 1000 μg/mL (*p* = 0.0001) of THD resulted in significantly decreased compared to controls, and had a similar inhibitory effect compared to 2.5 μM of BACE inhibitor (Figure 3E). Moreover, 5, 50, 500 and 1000 μg/mL (*p* = 0.0002) of THD significantly inhibited AchE activity compared to vehicle control, but it had no similar effect compared to 10uM of Donepezil (Figure 3E).

### 3.4. Attenuation of Lipid Uptake in Monocytes and Inhibition of Endothelial Cell Activation

**Figure 4:**
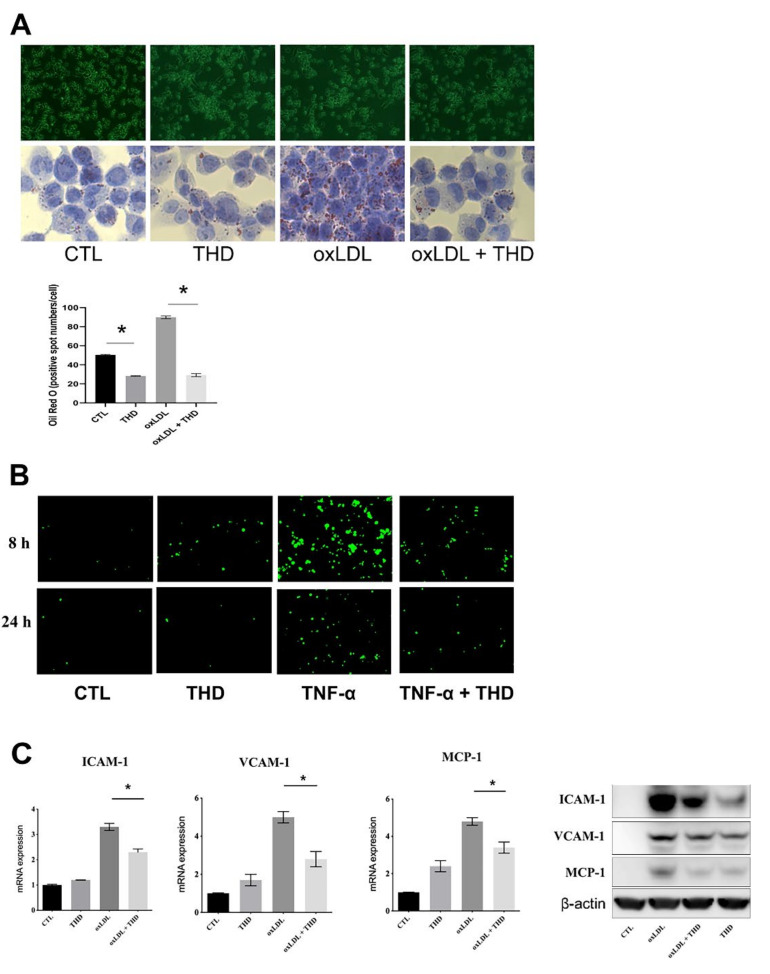
THD inhibits endothelial cell activation. (**A**) THP-1 cells were differentiated into macrophages and incubated with oxLDL (100 μg/mL) for 24 h, followed by Oil red O staining (*n* = 3). (**B**) oxLDL-stimulated HUVECs were incubated with fluorescently labeled THP-1 cells, followed by removal of unbound THP-1 cells (*n* = 3). (**C**) HUVECs were activated by oxLDL (100 μg/mL) treatment, and ICAM-1, VCAM-1, and MCP-1 expression levels were determined by qRT-PCR (left panel) and western blot (right panel) (*n* = 3). THD, Tongqiaohuoxue decoction; oxLDL, oxidized low-density lipoprotein; CTL, control; ICAM-1, intercellular adhesion molecule-1; VCAM-1, vascular cell adhesion molecule-1; MCP-1, monocyte chemoattractant protein-1. * *p* < 0.01 vs. corresponding control. *p* value was obtained from one-way ANOVA with Tukey’s post- hoc tests.

Activation of damaged endothelial cells is considered the priming event leading to formation of atherosclerotic plaques. Therefore, we used human umbilical vein endothelial cells (HUVEC) and THP-1 human monocytic cells to investigate the effect of THD on the activation of endothelial cells and the resultant clearance of lipid deposits in the aorta and brain vessels. The lipid uptake assay revealed that incubation of THP-1 cells with oxidized low-density lipoprotein (oxLDL) increased the number of cytosolic lipid droplets, while treatment with THD resulted in a clear reversal of the increased oxLDL uptake (Figure 4A). Adhesion assay analysis also showed decreased adhesion of THP-1 cells to HUVECs, indicating decreased activation of endothelial cells (Figure 4B).

To further validate an inhibitory effect of THD on endothelial cells activation, HUVECs were incubated with oxLDL (Figure 4C). Treatment with oxLDL increased intercellular adhesion molecule-1(ICAM-1), vascular cell adhesion molecule-1(VCAM-1), and monocyte chemoattractant protein-1(MCP-1) levels. However, THD mitigated the oxLDL-induced activation of HUVECs through downregulation of ICAM-1, VCAM-1, and MCP-1.

### 3.5. Reduction in Serum Resistin in ApoE-/- Mice and Resistin Expression of Human Monocyte

**Figure 5:**
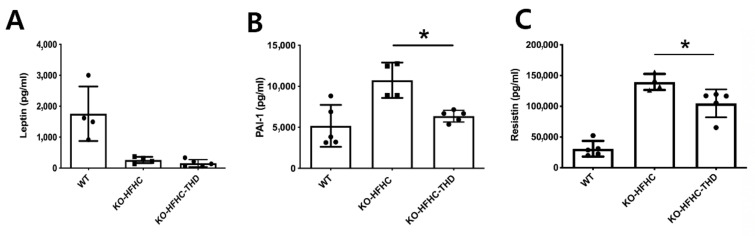
THD decreases serum levels of PAI-1 and resistin. The plasma levels of leptin (**A**), PAI-1 (**B**), and resistin (**C**) were analyzed by immunoassay analysis. WT-HFHC, wild type–high fat high cholesterol; KO-HFHC, ApoE-/- high fat high cholesterol; KO-HFHC-THD, ApoE-/- high fat high cholesterol–tongqiaohuoxue decoction. * *p* < 0.001 vs. KO-HFHC.

We next determined the serum levels of adipokines such as leptin, PAI-1, and resistin as contributing factors for damaged endothelial cells (Figure 5A–C). The levels of leptin, a prototype adipokine, were decreased in ApoE-/- mice, and THD treatment did not affect its levels in serum (Figure 5A). However, the increased serum levels of PAI-1 and resistin in ApoE-/- mice were decreased with THD treatment (Figure 5B,C).

**Figure 6:**
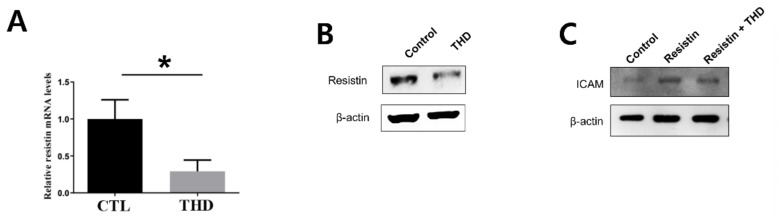
THD inhibits resistin expression and resistin-induced ICAM expression. THP-1 cells were differentiated, and the mRNA (**A**) and protein (**B**) expression levels of resistin were determined (*n* = 3). (**C**) HUVECs were activated by resistin treatment, and ICAM-1 expression levels were evaluated (*n* = 3). THD, Tongqiaohuoxue decoction; ICAM-1, intercellular adhesion molecule-1. * *p* < 0.001 vs. control.

In particular, human resistin is known to be an inflammatory marker of atherosclerosis and AD, dominantly expressed in macrophages [14] and leading to endothelial dysfunction through adhesion molecules. Thus, we assessed the effect of THD on the expression of resistin in the human macrophage cell line THP-1. Consistent with its inhibitory effect in ApoE-/- mice, THD inhibited the mRNA and protein expression levels of resistin in THP-1 cells (Figure 6A,B). Moreover, THD decreased the expression of resistin-induced ICAM-1 in HUVECs.

## 4. Discussion

In this study, we found that THD exerts therapeutic effects on both atherosclerosis and AD in HFHC-fed ApoE-/- mice. THD attenuated plaque formation and decreased lipid deposits in atherosclerotic arteries through inhibition of endothelial activation. It also decreased lipid deposition and plaque formation in the brains of ApoE-/- mice, suggesting a therapeutic effect on AD. Moreover, we observed that THD reduced the serum levels of resistin, a hormone known to be strongly linked to both atherosclerosis and AD.

Endothelial dysfunction, also known as endothelial activation, is considered to be the initiation process and an early marker of atherosclerosis [15]. The expression of adhesion molecules, such as ICAM (intercellular adhesion molecule) and VCAM (vascular cell adhesion molecule) is upregulated in activated endothelial cells, facilitating interactions with monocytes and leading to monocyte extravasation [16]. Our results support the theory that endothelial dysfunction and atherosclerosis may be involved in the pathogenesis of AD [17,18].

We verified the presence of the targeted 11 herbal compounds in our THD samples. Several have been shown to be active compounds that exert therapeutic effects on atherosclerosis. For example, amygdalin regulates the formation of atherosclerosis and stabilizes plaques by suppressing inflammatory responses and promoting the immune-modulatory function of T-regulatory cells [19]. Paeoniflorin suppresses the expression of ICAM-1 in lipopolysaccharide-treated U937 cells and HUVECs stimulated with tumor necrosis factor α [20]. In line with these results, we observed that THD reduces oxLDL-induced lipid deposits in and monocyte adhesion to HUVECs, indicating an inhibitory action on oxLDL-induced endothelial dysfunction. Furthermore, we validated this inhibitory action in endothelial cells by demonstrating decreased expression of ICAM-1, VCAM-1, and MCP-1 in THD-treated HUVECs.

ApoE is strongly associated with a high risk of atherosclerosis, the leading cause of cardiovascular disease characterized by lipid plaque formation in large vessels [21]. Human ApoE has three common isoforms: APOE2, APOE3, and APOE4. Individuals with the APOE4 allele show increased susceptibility to late-onset AD [22,23]. Moreover, APOE4 is closely associated with the integrity of tight junctions in a blood–brain barrier (BBB) mouse model, and ApoE deficiency in mice leads to BBB leakage [24].

Li et al. [25] reported that THD reduces the opening of tight junctions and decreases the permeability of the BBB by upregulating the expression of ZO-1, occludin, and claudin-5. In particular, the authors suggested that muscone, ligustilide, and safflomin A, which can pass through the BBB and are detectable in CSF, are the main active ingredients of THD that may attenuate the damaged BBB. In this study, safflomin A was also detected as the most abundant active compound in THD. However, to date, no published reports have examined whether THD is associated with the regulation of lipid deposition in the brain.

**Figure 7:**
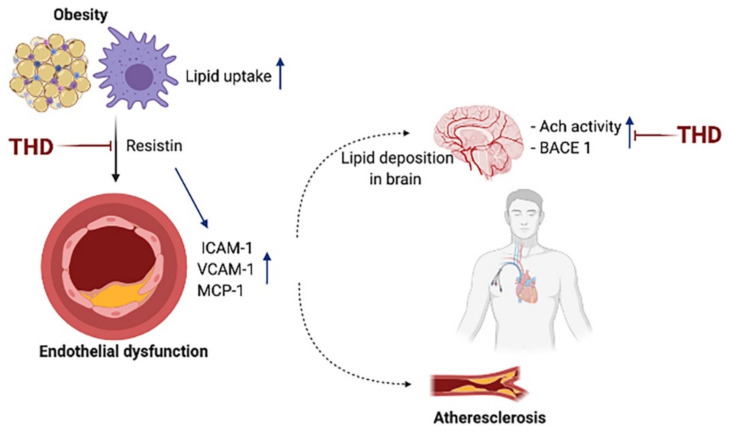
THD ameliorates endothelial activation possibly via inhibition of resistin. THD also decreases the accumulation of amyloid plaque possibly via inhibition of ß-secretase (BACE 1) and acetylcholinesterase (Ach) activities.

In the current study, we first assessed lipid deposition in the ChP, the region of the primary intracranial neuroimmunological interface that establishes the blood–CSF barrier. The ChP constitutes a major barrier to the penetration of blood-borne leukocytes into the central nervous system; this role may be compromised by lipid deposition in inflammatory and degenerative brain diseases [26,27,28,29,30]. In line with a previous report [8], we observed increased depositions of lipid and amyloid plaque formation in the ChP region in ApoE-/- mice. We also found that THD abrogates HFHC-induced amyloid plaque formation and its related enzymes, and lipid deposition in the ChP, unravelling the inhibitory effect of THD on lipid deposition-related brain abnormalities. In addition, these results may be associated with a protective effect of BBB and suppression of lipid uptake by monocytes (Figure 7).

Notably, in addition to the decreased production of PAI-1, we showed that THD decreases serum resistin levels not only in ApoE-/- mice but also in THP-1 cells. The major secretion origin and function of resistin differ between mouse and human. In rodents, resistin is secreted from mature adipocytes and involved in obesity-related diseases. In contrast, human resistin is primarily secreted from monocytic cells, thus recruiting other immune cells and activating the secretion of pro-inflammatory factors [31]. Increasing evidence links human resistin to inflammation and atherogenesis [32,33]. Here, we also validated that resistin induces the expression of ICAM-1 in HUVECs, and that THD exerts its anti-atherosclerotic effects by regulating resistin both in a rodent model and in a human cell line. Considering that resistin is a hormone with important roles both in AD [34] and in atherosclerosis [33] these results support a therapeutic effect of THD on both diseases (Figure 7). In addition, multi-cohort and clinical studies have reported that resistin is associated with an increased risk of AD [35,36,37].

## 5. Conclusions

Here, we uncovered the evidence of novel anti-inflammatory functions of THD in the development and progression of AD and atherosclerosis. However, further studies are needed to determine the bioactive compounds and their underlying molecular mechanisms in exerting the THD effects on AD and atherosclerosis, in order to reduce overcome the limitation of natural crude extracts. Notwithstanding, our results support the need for further investigations into the mechanisms and potential clinical applications of THD in the treatment of AD and atherosclerosis. In addition, this preliminary study highlights the potential benefits of THD, possibly via inhibition of resistin-mediated activation of endothelial cells and BACE1 and AChE-mediated accumulation of amyloid plaques, for both conditions.

## Supplementary Materials

The following are available online at https://www.mdpi.com/2079-7737/9/11/363/s1, Table S1. Full scientific names of herbal plants in Tongqiaohuoxue decoction. Table S2. Linear range, regression equation, *r*^2^, limit of detection (LOD), and limit of quantification (LOQ) for the 12 marker compounds (*n* = 3). Table S3. Amounts of the 11 marker components in THD sample by HPLC (*n* = 3).
