# Supplementary material for: Tongqiaohuoxue Hinders Development and Progression of Atherosclerosis: A Possible Role in Alzheimer’s Disease
Source: Biology (Basel). 2020 Oct 27;9(11):363. doi: 10.3390/biology9110363 (PMC7692730; doi:10.3390/biology9110363)
Supplement: Supplementary file 1 [file biology-09-00363-s001.pdf]

Table S1. Full scientific names of herbal plants in Tongqiaohuoxue decoction.

Number	Common name (Korea Pharmacopoeia)	Plant full scientific name (Kew MPNS)
1	*Paeoniae Radix*	*Paeonia obovata* Maxim.
2	*Cnidii Rhizoma*	*Ligusticum officinale* (Makino) Kitag.
3	*Persicae Semen*	*Prunus persica* (L.) Batsch
4	*Carthami Flos*	*Carthamus tinctorius* L.
5	*Allii Fistulosi Bulbus*	*Allium fistulosum* L.
6	*Zizyphi Semen*	*Ziziphus jujuba* Mill.
7	*Zingiberis Rhizoma*	*Zingiber officinale* Roscoe
8	*Aucklandiae Radix*	*Aucklandia costus* Falc.

Table S2. Linear range, regression equation, r^2, limit of detection (LOD), and limit of quantification (LOQ) for the 12 marker compounds (n = 3).

Compound	Linear range (μg/mL)	Regression equation (y = ax + b)[a]	r^2	LOD (μg/mL)[b]	LOQ (μg/mL)[c]
Gallic acid	0.31–20.00	$y = 38248.11x - 763.69$	0.9999	0.04	0.13
Amygdalin	1.56–100.00	$y = 7045.32\,x + 3465.97$	0.9999	0.50	1.51
Albiflorin	0.31–20.00	$y = 13028.12x - 545.93$	1.0000	0.04	0.12
Paeoniflorin	1.56–100.00	$y = 13991.49x - 5786.07$	0.9999	0.16	0.48
Ferulic acid	0.31–20.00	$y = 69067.29x - 992.60$	0.9999	0.02	0.07
Safflomin A	3.13–200.00	$y = 33948.36x - 15212.56$	0.9999	0.49	1.48
Benzoic acid	0.31–20.00	$y = 60402.66x + 1765.32$	1.0000	0.07	0.20
Benzoylpaeoniflorin	0.31–20.00	$y = 21232.87x + 5269.96$	0.9999	0.06	0.17
6-Gingerol	0.31–20.00	$y = 6899.57x + 7.48$	0.9999	0.05	0.15
Costunolide	0.31–20.00	$y = 5077.84x - 279.53$	0.9999	0.10	0.30
Dehydrocostus lactone	0.31–20.00	$y = 16889.37x + 3344.47$	0.9999	0.07	0.21

[a] y: peak area (mAU) of compounds; x: concentration (μg/mL) of compounds. [b] LOD = 3.3 × σ/S, [c] LOQ = 10 × σ/S, Where, σ is standard deviation of the y-intercept and ach calibration curve (σ) and S is the slope of the calibration curve.

Table S3. Amounts of the 11 marker components in THD sample by HPLC (n=3).

Compound	Mean (mg/g)	SD × 10^{-2}	RSD (%)
Gallic acid	0.41	0.17	0.42
Amygdalin	3.64	10.09	2.77
Albiflorin	0.13	0.23	1.83
Paeoniflorin	2.95	1.94	0.66
Ferulic acid	0.25	0.12	0.48
Safflomin A	7.44	0.28	0.04
Benzoic acid	0.33	0.06	0.18
Benzoylpaeoniflorin	0.09	0.13	1.47
6-Gingerol	0.08	0.05	0.66
Costunolide	0.28	0.25	0.89
Dehydrocostus lactone	0.14	0.04	0.30
